# Allometric scaling patterns among the human coronary artery tree, myocardial mass, and coronary artery flow

**DOI:** 10.14814/phy2.14514

**Published:** 2020-07-29

**Authors:** Jin‐Ho Choi, Eunsoo Kim, Hyung Yoon Kim, Seung‐Hwa Lee, Sung Mok Kim

**Affiliations:** ^1^ Department of Emergency Medicine Samsung Medical Center Sungkyunkwan University School of Medicine Seoul Republic of Korea; ^2^ College of Life Science and Biotechnology Korea University Seoul Republic of Korea; ^3^ Department of Cardiovascular Medicine Chonnam National University Hospital Gwangju Republic of Korea; ^4^ Department of Medicine Samsung Medical Center Sungkyunkwan University School of Medicine Seoul Republic of Korea; ^5^ Depart of Radiology Samsung Medical Center Sungkyunkwan University School of Medicine Seoul Republic of Korea

**Keywords:** allometric scaling, coronary artery, imaging and physiology

## Abstract

Human coronary artery tree is a physiological transport system for oxygen and vital materials through a hierarchical vascular network to match the energy demands of myocardium, which has the highest oxygen extraction ratio among body organs and heavily depends on the blood flow for its energy supply. Therefore, it would be reasonable to expect that the key design principle of this arterial network is to minimize energy expenditure, which can be described by allometric scaling law. We enrolled patients who underwent coronary computed tomography angiography without obstructive lesion. The cumulative arterial length (*L*), volume (*V*), and diameter (*D*) in relation to the artery‐specific myocardial mass (*M*) were assessed. Flow rate (*Q*) was computed using quantitative flow ratio (QFR) measurement in patients who underwent invasive angiography. A total of 638 arteries from 43 patients (mean age 61 years, male gender 65%) were analyzed. A significant power‐law relationship was found among *L*–*M*, *V*–*M*, *D*–*M*, *V*–*L*, *D*–*L*, and *V*–*D*, and also among *Q*–*M*, *Q*–*L*, *Q*–*V*, and *Q*–*D* in 106 arteries interrogated with QFR (*p* < .001, all). Our results suggest that the fundamental design principle of the human coronary arterial network may follow allometric scaling law.

## Graphical abstract

Allometric scaling law was demonstrated among myocardial mass, vessel length, vessel volume, and diameter of human coronary artery.

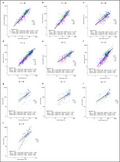

## 1 INTRODUCTION

Coronary artery tree is a physiological transport system for oxygen and vital materials into the capillary bed through a hierarchical vascular network to match myocardial demands (Schelbert, 2010). Myocardium is a biological pump that relies almost exclusively on the aerobic oxidation of substrate for its mechanical work (Knaapen et al., 2007). Furthermore, myocardium has the highest oxygen extraction ratio among all body organs and heavily depends on the blood flow for its energy supply (Hoffman & Buckberg, 2014). The mechanical efficiency of myocardium is estimated to be about 25%, and it decreases further in pathophysiological status such as heart failure (Ingwall, 2008). Therefore, it would be reasonable to expect that the design principle of coronary artery tree is to provide blood flow with maximal efficacy (Huo & Kassab, 2009a, 2012; Kassab & Finet, 2015). Under this hypothesis, the geometry of arterial tree would form a hierarchical network that obeys a set of scaling patterns to fulfill energy‐efficient transport of materials through the arterial tree (Huo & Kassab, 2009b).

The size or function of many organs is known to be proportional to the quarter‐power scaling of the organ mass (West et al., 1997). This allometric scaling model has successfully described how morphological or functional parameters of living organism change with scale (Darveau, Suarez, Andrews, & Hochachka, 2002; Goldbogen et al., 2019; White & Seymour, 2003). Allometric scaling also explains why the geometry of the arterial tree forms a hierarchical network (Kassab, 2006). It is the result of interplay among minimizing the cost of space occupied by the hierarchical network and maximizing the transport with the least amount of energy loss through the network (Chen et al., 2015). Using this concept, we can transform the heterogenous sizes and branching patterns of coronary artery tree into a simple elegant mathematical model.

Allometric scaling in coronary artery tree was previously investigated in animal models, but there are limited studies on the human heart (Chen et al., 2015; Seiler, Kirkeeide, & Gould, 1993; Zhou, Kassab, & Molloi, 1999, 2002). We investigated the scaling patterns among morphological and physiological parameters in the human coronary artery tree and myocardium.

## 2 METHODS

### 2.1 Patients

We retrospectively enrolled patients who underwent clinically indicated coronary computed tomography (CT) angiography and were found to have no obstructive lesion from January 2016 to August 2018. Patients with prior history of acute coronary syndrome, revascularization, heart failure, valvular heart disease, or complex structural or congenital heart disease were not included. Invasive coronary angiography was performed in selected patients for screening of variant angina. No vasospasm was reported. The study protocol was approved by the institutional review board of Samsung Medical Center.

### 2.2 Coronary CT angiography

**FIGURE 1:**
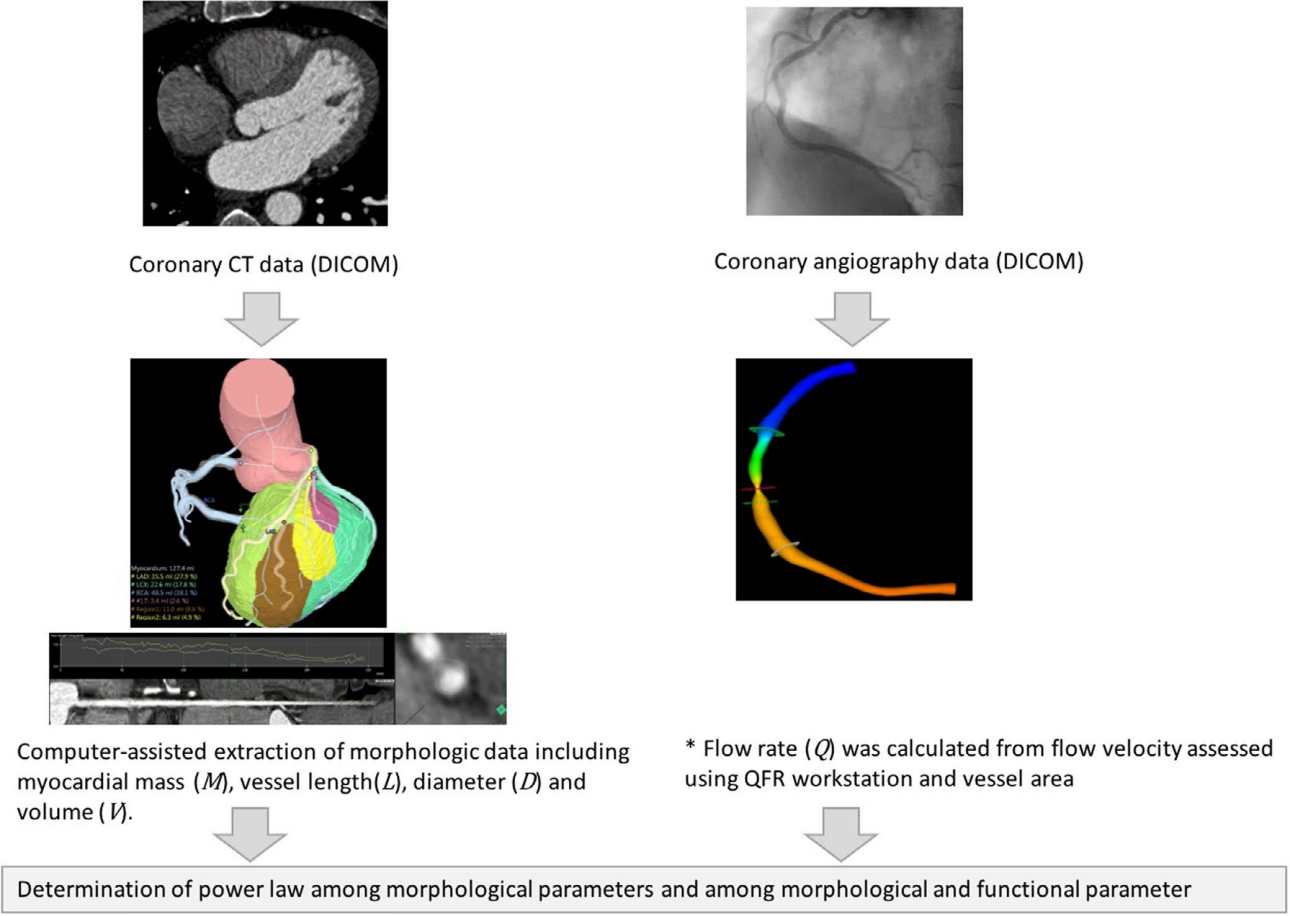
From coronary CT data, the cumulative arterial tree length (*L*), cumulative arterial tree volume (*V*), and arterial proximal diameter (*D*) of each segment were measured. Segment‐specific left ventricular myocardial mass (*M*) was measured using a dedicated software module in Fujifilm Synapse Vincent workstation. Coronary blood flow (*Q*) was calculated from flow velocity assessed from quantitative flow ratio (QFR) software (QAngio XA 3D) and vessel area. Then, a scaling model of the form was fitted to the dataset where X and Y are one of *M*, *L*, *V*, *D*, or *Q*. * QFR was assessed in 95 vessels

The scheme of the study flow is summarized in Figure 1. Coronary CT angiography was performed using multivendor CT scanners equipped with 64‐slice or higher detectors as described previously (Kim et al., 2016). Sublingual nitroglycerin of 0.6 mg was prescribed to each patient before scanning. The image data set was reconstructed using 0.5 or 0.6 mm slices. A dedicated CT workstation (Fujifilm Synapse Vincent, Tokyo, Japan) was used by two experienced imaging specialists who were blinded to patient data. Three‐dimensional coronary arterial tree model was constructed and segmented according to the modified American Heart Association classification. All major epicardial coronary arteries and first‐order branches ≥1.0 mm in diameter were tracked from the ostium to the distal end. Arterial central axis was determined with assistance of automatic tracking function and was confirmed by reviewing cross sections. Then arterial segments that do not directly perfuse left ventricular myocardium; right coronary artery (RCA) segment from ostium to distal RCA, right ventricular branches, and left main segment; were excluded as described previously (Kim et al., 2016). The cumulative arterial tree length (*L*), cumulative arterial tree volume (*V*), and arterial proximal diameter (*D*) of each segment were measured. Segment‐specific left ventricular myocardial mass (*M*) was measured using a dedicated software module based on Voronoi tessellation (Murai et al., 2019).

### 2.3 Invasive coronary angiography and quantitative flow ratio (QFR)

Invasive coronary angiography was performed according to the standard protocol. After intracoronary nitroglycerin injection, two angiographic images with optimized projections were obtained at 15 frame/sec for each major coronary artery. Contrast medium was injected by automated injection pump at a rate of approximately 4 ml/sec.

Off‐line quantitative flow ratio (QFR) analysis was performed using QAngio XA 3D (Medis, Leiden, the Netherlands) by a certificated trained technician who was blinded to any other imaging or clinical data. In brief, the calculation of QFR is based on the three‐dimensional reconstruction of coronary angiograms from two projections with angles ≥25 degree apart and volumetric flow rate assessed by angiographic frame count (Tu et al., 2016). The blood flow velocity and arterial diameter of left anterior descending artery (LAD) and left circumflex artery (LCX) assessed using QFR were used to calculate flow rate (*Q*). QFR was not used in RCA due to the intraprocedural motion, change in projection angle, and different coronary flow pattern of RCA compared to LAD and LCX (Hadjiloizou et al., 2008).

### 2.4 Calculation of scaling pattern and statistical analysis

A scaling model of the form *Y* = *Y*_0_ *X^b^* was fitted to the dataset where *X* and *Y* are one of *M*, *L*, *V*, *D*, or *Q*, *Y*_0_ is a normalization constant, and *b* is the power‐law exponent. The results were logarithmically transformed with log *Y* = *b* log *X* + log *Y*_0_ format adjusted for individual and were shown in log‐log scatter plots.

Ordinary least square regression is the most popular method for linear regression. It minimizes the sum of squared residuals in Y‐axis values and calculates *r*‐squared coefficient and statistical *p*‐value. However, it is based on the premise of accurate measurement in X‐axis values and constant variance in Y‐axis values, which may not be met in real‐world dataset, and it is highly sensitive to outliers. Therefore, Deming regression that counts for distances in both X‐ and Y‐axis values, and Theil‐Sen estimator that uses the median of all slope lines through pairs of points and is less affected by outliers, were additionally calculated (Ghosh, 2008). The results of ordinary least square, Deming, and Theil‐Sen were shown using mean ± *SE*, mean with 95% confidence interval (CI), and median with interquartile range (IQR), respectively. For ordinary least square regression, both unadjusted and adjusted for individual results were reported. The difference of slope among LAD, LCX, and RCA plot was assessed from Tukey's honest significant differences adjusted for individual. All analyses were performed with R version 3.6 (R foundation). A two‐tailed *p* < .05 was considered to be significant.

## 3 RESULTS

### 3.1 Patients

Clinical characteristics of 43 patients are shown in Table 1. A total of 638 arteries was analyzed using CT workstation. Invasive coronary angiography was performed in 19 patients without intervening cardiovascular event with median interval of 24 days. A total of 106 arteries were interpretable by QFR software.

**TABLE 1:** Clinical characteristics

*N*	43
Age (year)	61 ± 13
Male gender	28 (65.1)
Body mass index (kg/m^2^)	23.9 ± 2.5
Body surface area (m^2^)	1.71 ± 0.16
Hypertension	7 (16.3)
Diabetes	4 (9.3)
Hyperlipidemia	7 (16.3)
Smoking	6 (14.0)
Systolic blood pressure (mmHg)	122 ± 14
Diastolic blood pressure (mmHg)	74 ± 15
Hemoglobin (g/dl)	13.4 ± 2.1
Creatinine (mg/1)	1.2 ± 1.9
Total cholesterol (mg/dl)	165 ± 35
LDL cholesterol (mg/dl)	97 ± 31
HDL cholesterol (mg/dl)	56 ± 26
Triglyceride (mg/dl)	146 ± 95
Left ventricular mass (g)	129 ± 44
Left ventricular mass index (g/m^2^)	75 ± 24

### 3.2 Scaling patterns

**FIGURE 2:**
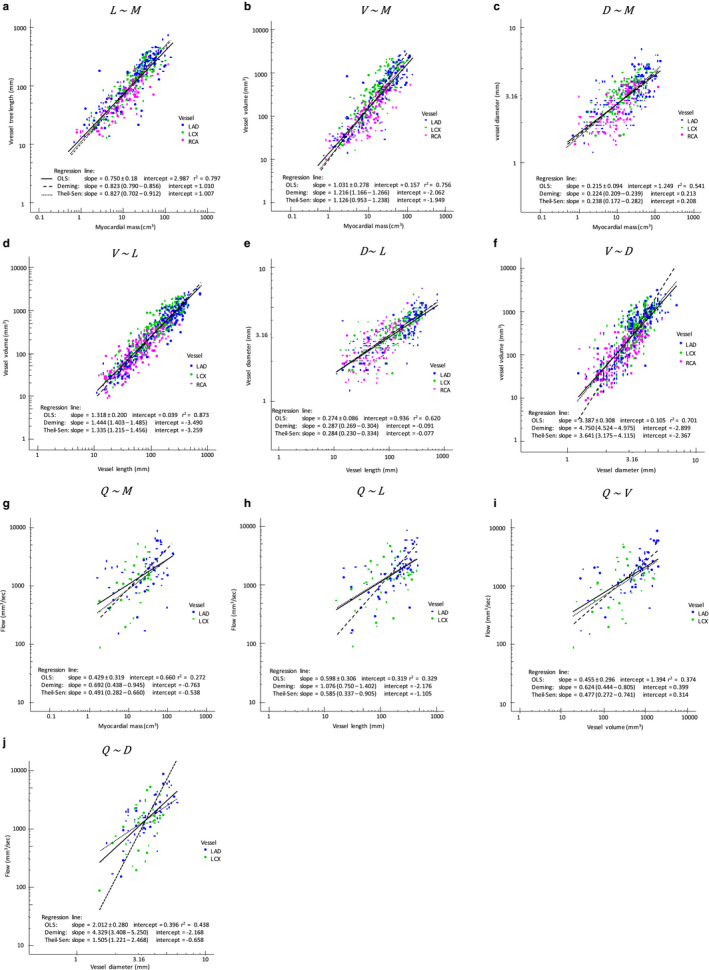
Log‐log plots among segment‐specific left ventricular myocardial mass (*M*), cumulative arterial tree length (*L*), cumulative arterial tree volume (*V*), arterial proximal diameter (*D*), blood flow (*Q*) are shown. LAD, LCX, and RCA are shown using color scheme. Blood flow was not assessed in RCA. Regression lines of ordinary least square (OLS), Deming, and Theil‐Sen methods are shown using solid, crudely dotted, and fine dotted lines, respectively

The morphological and flow characteristics are summarized in Table 2. Scaling patterns among morphological and functional parameters are shown in log‐log plots. Overall, significant power‐law relationships were found among all parameters. The exponents of ordinary least square regressions were 0.795 ± 0.140 between *L* and *M*, 1.080 ± 0.229 between *V* and *M*, 0.224 ± 0.082 between *D* and *M*, 1.322 ± 0.175 between *V* and *L*, 0.277 ± 0.075 between *D* and *L*, 3.487 ± 0.287 between *V* and *D*, 0.498 ± 0.273 between *Q* and *M*, 0.635 ± 0.267 between *Q* and *L*, 0.486 ± 0.259 between *Q* and *V*, and 2.271 ± 0.235 between *Q* and *D* (*p* < .001, all, Figure 2). The exponents of ordinary least square regression as well as Deming regression and Theil‐Sen estimator were grossly consistent in three major coronary arteries including LAD, LCX, and RCA as shown in colored scheme in Figure 2 and summarized in Table 3. In a comparison of slope among LAD, LCX, and RCA, statistically significant difference was noted in the exponents of correlation except V–*D*, *Q*–*L*, *Q*–*V*, and *Q*–*D* (Table 4).

**TABLE 2:** Morphological and flow characteristics of vessel

Per‐vessel data (*N* = 638)	
Vessel location	
LAD	233 (36.6)
LCX	209 (32.8)
RCA	196 (30.7)
Vessel diameter (mm)	3.10 ± 0.97
Vessel area (mm^2^)	8.30 ± 5.18
Vessel tree length (mm)	132 ± 113
Myocardial mass (cm^3^)	23.0 ± 21.9
Vessel volume (mm^3^)	437 ± 501
Per‐vessel data, flow assessed (*N* = 106)	
Flow velocity (mm/sec)	182 ± 109
Flow rate (mm^3^/sec)	1907 ± 1529
Per‐patient data (*N* = 43)	
Total vessel volume (mm^3^)	2,410 ± 941
Total myocardial mass (cm^3^)	129 ± 45

Note

LAD, left anterior descending artery; LCX, left circumflex artery; RCA, right coronary artery

**TABLE 3:** Correlation coefficients

		Ordinary least square				Demming		Theii‐Sen	
		*b*	*Y*o	*r* ^2^	*p*	*b*	*Y*_0_	*b*	*Y*_0_
*L*–*M*	Unadjusted	0.750 ± 0.180	2.987	0.797	<.001	0.823 (0.790–0.856)	1.010	0.827 (0.702–0.912)	1.007
	Adjusted for individual	0.795 ± 0.140	3.010	0.877	<.001	—	—	—	—
*V*–*M*	Unadjusted	1.031 ± 0.278	0.157	0.756	<.001	1.216 (1.166–1.266)	−2.062	1.126 (0.953–1.238)	−1.949
	Adjusted for individual	1.080 ± 0.229	2.925	0.835	<.001	—	—	—	—
*D*–*M*	Unadjusted	0.215 ± 0.094	−1.249	0.541	<.001	0.224 (0.209–0.239)	0.213	0.238 (0.172–0.282)	.208
	Adjusted for Individual	0.224 ± 0.082	1.209	0.665	<.001	—	—	—	—
*V*–*L*	Unadjusted	1.318 ± 0.200	0.039	0.873	<.001	1.444 (1.403–1.485)	−3.490	1.335 (1.215–1.456)	−3.259
	Adjusted for individual	1.322 ± :0.175	0.701	0.903	<.001	—	—	—	—
*D*–*L*	Unadjusted	0.274 ± 0.086	0.936	0.620	<.001	0.287 (0.269–0.304)	0.091	0.284 (0.230–0.334)	−.077
	Adjusted for individual	0.277 ± 0.075	0.895	0.713	<.001	—	—		—
*V*–*D*	Unadjusted	3.387 ± 0.308	0.105	0.701	<.001	4.750 (4.524–4.975)	−2.899	3.641(3.175–4.115)	−2.367
	Adjusted for individual	3.487 ± 0.287	2.040	0.739	<.001	—	—	—	—
*Q*–*M*	Unadjusted	0.429 ± 0.319	0.660	0.272	<.001	0.692 (0.438–0.945)	0.763	0.491(0.282–0.660)	−.538
	Adjusted for individual	0.498 ± 0.273	10.293	0.467	<.001	—	—	—	—
*Q*–*L*	Unadjusted	0.598 ± 0.306	0.319	0.329	<.001	1.076 (0.750–1.402)	−2.176	0.585 (0.337 –0.905)	−1.105
	Adjusted for individual	0.635 ± 0.267	4.683	0.489	<.001	—	—	—	—
*Q*–*V*	Unadjusted	0.455 ± 0.296	1.394	0.374	<.001	0.624 (0.444–0.805)	0.399	0.477 (0.272 –0.741)	.314
	Adjusted for individual	0.486 ± 0.259	5.483	0.521	<.001	—	—	—	—
*Q*–*D*	Unadjusted	2.012 ± 0.280	0.396	0.438	<.001	4.329 (3.408– 5.250)	−2.168	1.505 (1.221–2.468)	−.658
	Adjusted for individual	2.271 ± 0.235	5.779	0.605	<.001	—	—	—	—

Note

The exponent (*b*) and coefficient (*Y*_0_) of scaling law among segment‐specific myocardial mass (*M*), cumulative arterial tree length (*L*), cumulative arterial tree volume (*V*), arterial proximal diameter (*D*), and blood flow (*Q*). *b* represents mean ± SE in ordinary least square, mean, and 95% confidence interval in Deming, and median with interquartile range in Theil‐Sen.

**TABLE 4:** Correlation coefficients among LAD, LCX, and RCA

	Vessel	*b*	*Y*_o_	*r* ^2^	Comparison	Tukey's honest significant differences	*p*‐value
*L*–*M*	LAD	0.746 ± 0.18	3.111	.830	LAD versus LCX	−0.022	.29
	LCX	0.795 ± 0.149	2.970	.082	LAD versus RCA	0.092	<.001
	RCA	0.625 ± 0.164	3.088	.739	LCX versus RCA	0.114	<.001
*V*–*M*	LAD	1.020 ± 0.277	3.248	.795	LAD versus LCX	−0.098	<.001
	LCX	1.121 ± 0.234	3.234	.787	LAD versus RCA	0.071	.002
	RCA	0.829 ± 0.224	3.313	.728	LCX versus RCA	0.169	<.001
*D*–*M*	LAD	0.214 ± 0.094	1.255	.595	LAD versus LCX	−0.077	.015
	LCX	0.216 ± 0.083	1.278	.523	LAD versus RCA	−0.026	.64
	RCA	0.192 ± 0.098	1.244	.429	LCX versus RCA	0.051	.18
*V*–*L*	LAD	1.337 ± 0.177	0.732	.916	LAD versus LCX	−0.070	<.001
	LCX	1.329 ± 0.195	0.822	.851	LAD versus RCA	−0.031	.031
	RCA	1.169 ± 0.208	0.979	.766	LCX versus RCA	0.039	.006
*D*–*L*	LAD	0.277 ± 0.085	0.924	.699	LAD versus LCX	−0.050	.045
	LCX	0.254 ± 0.079	0.985	.559	LAD versus RCA	−0.119	<.001
	RCA	0.287 ± 0.091	0.912	.504	LCX versus RCA	−0.069	.005
*V*–*D*	LAD	3.560 ± 0.309	1.990	.745	LAD versus LCX	−0.003	.88
	LCX	3.367 ± 0.307	2.254	.633	LAD versus RCA	0.004	.81
	RCA	2.617 ± 0.263	2.603	.626	LCX versus RCA	0.008	.54
*Q*–*M*	LAD	0.328 ± 0.318	15.686	.181	LAD versus LCX	−0.157	.034
	LCX	0.586 ± 0.307	10.605	.364			
*Q*–*L*	LAD	0.610 ± 0.276	6.487	.383	LAD versus LCX	−0.031	.59
	LCX	0.501 ± 0.343	7.343	.206			
*Q*–*V*	LAD	0.461 ± 0.265	7.302	.433	LAD versus LCX	−0.038	.62
	LCX	0.393 ± 0.335	7.831	.242			
*Q*–*D*	LAD	1.937 ± 0.249	8.585	.501	LAD versus LCX	−0.005	.78
	LCX	1.961 ± 0.322	7.690	.303			

Note

Tukey's honest significant differences adjusted for individual are shown.

## 4 DISCUSSION

The major finding of this study was a demonstration of allometric scaling relations among myocardial mass, artery, and flow in human heart. Our study is one of the first to show a power‐law relationship among morphological and functional parameters in the human coronary circulation.

### 4.1 Allometric scaling in coronary artery morphology

The scaling exponents of allometric scaling were variable in prior studies. For example, the scaling exponent of *V*–*L* ranged from 0.71 to 0.80 (Kassab, 2006; Le, Wong, & Molloi, 2008; Mittal et al., 2005; Wischgoll, Choy, & Kassab, 2009). Kassab et al. reported in their mathematical and animal model studies that the scaling exponent *b* among myocardial mass and coronary artery morphology as follows: *L*–*M*, *V*–*M*, *D*–*M*, *V*–*L*, *D*–*L*, *V*–*D*, *Q*–*M*, and *Q*–*D* was 3/4, 1, 3/8, 9/7, 3/7, 3, 3/4, and 7/3, respectively (Choy & Kassab, 2008; Huo & Kassab, 2009a). In our study, the scaling exponents of *L*–*M* (*b* = 0.750, difference = 0%), *V*–*M* (*b* = 1.031, difference = 3.1%), and *V*–*L* (*b* = 1.318, difference = 2.4%) differed from the theoretical values by 0 to 3%. The scaling exponents of *D*–*M* (*b* = 0.215, difference = −42.7%) and *D*–*L* (*b* = 0.274, difference = −36.0%) were lower than the theoretical values, and the exponent for *V*–*D* (*b* = 3.387, difference = 12.9%) was higher than the theoretical values. These variations may be reasonably explained by the methodological limitation in the range of scaling. In our human study, the hierarchy of allometric scaling was limited to epicardial arteries. Small arteries and capillary network were not included in the calculation of scaling exponent. Regression analysis of a truncated portion may be not identical to analysis of the whole‐range arterial tree (Savage, Deeds, & Fontana, 2008).

In the comparison among vessels, RCA showed smaller arterial length, arterial volume, and diameter given the same size of myocardium. The less attenuated diastolic flow pattern in RCA compared to LAD or LCX may contribute to the smaller arterial size of RCA for myocardium (Goodwill, Dick, Kiel, & Tune, 2017; Lee & Smith, 2012).

### 4.2 Allometric scaling in coronary artery flow

Unlike the scaling exponents of arterial size, the scaling exponents of flow rate including *Q*–*M* (*b* = 0.429, difference = −42.8%) and *Q*–*D* (*b* = 2.012, difference = −13.8%) were much lower than the theoretical values. The complex physiological regulation of coronary circulation may be one of the underlying causes of the apparently lower scaling exponents of flow rate. Coronary blood flow highly depends on the microvascular network which has arterial size below the spatial limitation of coronary CT, and it can increase by two‐ to threefold compared to the resting status in response to the dilatation or recruitment of microvascular network, which is known as coronary or myocardial flow reserve. Coronary artery system may be designed to provide the most energy‐efficient blood flow transport not at resting status but at maximal coronary vasodilatory status. If so, the scaling exponents for flow rate can be underestimated compared to the theoretical value in the resting status (Gould & Johnson, 2018). The scaling exponent would be also affected by the rheological property of blood in microvascular network, which consists of viscoelastic vessels covered with endothelial lining that interacts dynamically with red blood cells, white blood cells, platelets, and macromolecules such as fibrinogen. The regional perfusion heterogeneity caused by shear force‐mediated reversible aggregation of red blood cells and vessel size‐dependent change in blood viscosity known as the Fahraeus‐Lindqvist effect may also affect the scaling exponent (Popel & Johnson, 2005; Pries, Neuhaus, & Gaehtgens, 1992; Pries & Reglin, 2017).

Fixed dose of nitroglycerin was used in this study. The higher was the dose of vasodilator, the greater was the extent of vasodilation, which may significantly change the diameter, luminal volume, and the flow of the coronary artery. Therefore, achievement of maximal coronary vasodilatation is critical for the measurement of coronary artery dimension. The theoretically ideal method for maximal vasodilation is the use of incremental dose of single or multiple vasodilators until vasodilatation reaches a stationary response without further flow increase with an additional dose. Such a methodology is not practical in clinical study because of increased risk of hypotension, reflex tachycardiac, atrioventricular block, or severe chest pain. There are also the other confounding factors such as individual variation in endothelial function, density of capillary network, and subclinical atherosclerosis that can be identified only using intravascular ultrasound or optical coherence tomography (Adjedj et al., 2015; Alexopoulos et al., 2016).

The exponents derived from Deming regression and Theil‐Sen estimator showed slightly higher values than those of ordinary least square regression. Savage et al. suggested that the actual value of 3/4‐power scaling among metabolic rate and body mass known as West, Brown, and Euquist model depends on the body size and may have a higher value of 0.81 (Savage et al., 2008). Further studies are required to investigate the impact of perfusion heterogeneity, flow reserve, or size‐dependent value of exponents on the allometric scaling in coronary artery circulation.

### 4.3 Allometric scaling in the pathophysiology of coronary artery disease and left ventricular myocardium

The concept of allometric scaling suggests that ‘normal coronary artery’ or ‘normal myocardial mass’, which has been defined empirically, can be defined scientifically and strictly. Given the size of a myocardium, the most optimal size of an artery to conduct blood flow with minimal energy loss or the lowest pressure drop can be defined and vice versa.

Current diagnosis of coronary artery disease largely depends on the relative percent stenosis of vessel lumen. Allometric scaling law has been used successfully to afford morphological determination of physiological stenosis and the amount of myocardial mass subtended by stenosis (Bae et al., 2018; Kim et al., 2016, 2017; Murai et al., 2019). Allometric scaling law may provide a rationale for definition of left ventricular hypertrophy as well as a rationale for quantitative diagnosis of diffuse coronary artery disease, inappropriately small arterial luminal volume, or diabetic coronary artery disease (Huo et al., 2013; Taylor et al., 2017). Left ventricular hypertrophy is linked to adverse outcome by the degree of severity, and it can be regressed by mechanical unloading or medical treatment (Ali et al., 2011; Fagard, Celis, Thijs, & Wouters, 1979). The normal value of left ventricular mass has been derived from general population and adjusted by body surface area. With the advent of computational mechanics and flow dynamics, the concept of allometric scaling may facilitate scientific redefining of normal left ventricular mass.

The concept of allometric scaling is intuitive to physician and can be simply stated as “form serves function, and function influences form.” With the concept of allometric scaling, the aim in the clinical decision‐making process may be more focused on the efficacy of coronary circulation rather than simple measurement of size or stenosis.

### 4.4 Limitations

We enrolled patients without obstructive coronary artery disease. However, the spatial resolution of current coronary CT is limited in the detection of mild diffuse atherosclerosis, which might contribute to these vessel diameter, decreased flow rate, and larger vessel volume‐to‐diameter ratio in our study compared to the theoretically provided values (Huo et al., 2013). The diameter and volume of coronary artery may decrease in case of coronary vasospasm or incomplete vasodilatation. Coronary CT image was acquired with use of sublingual nitroglycerin and intravenous administration of contrast dye to minimize the risk of vasospasm. The flow rate was assessed indirectly from angiographic image analysis using QFR. However, accurate measurement of flow rate needs dedicated invasive devices such as intracoronary Doppler wire or thermodilution catheter (Pijls et al., 2002). The hierarchy of allometric scaling was limited to epicardial arteries. The density or heterogeneity of capillary network density not matching the scaling law may affect the perfusion and flow rate, but was not interrogated in this study (Savage et al., 2008).

## 5 CONCLUSIONS

This study showed allometric scaling patterns among myocardial mass, coronary artery size, and coronary blood flow in human coronary circulation. It provides insight into the fundamental design principle of human coronary circulation and may facilitate scientific definition of coronary pathophysiology such as diffuse coronary artery disease or left ventricular hypertrophy. Further studies with various extents of coronary artery disease or left ventricular hypertrophy are warranted to apply the concept of allometric scaling in clinical medicine.

## CONFLICTS OF INTEREST

None declared.

## AUTHOR CONTRIBUTIONS

J.C. and H.K. conceived and designed the research. All authors performed experiments, analyzed and interpreted data, and drafted manuscript. All authors edited, revised, and approved the final version of the manuscript.
